# Supplementary material for: Dietary Flavonoid Intakes in France Are Linked to Brewed Tea Consumption and to Socioeconomic Status: Analyses of the Third French Individual and National Food Consumption (INCA3) Survey for Children and Adults
Source: Nutrients. 2024 Apr 10;16(8):1118. doi: 10.3390/nu16081118 (PMC11054843; doi:10.3390/nu16081118)
Supplement: Supplementary file 1 [file nutrients-16-01118-s001.zip › nutrients-2889480-supplementary.pdf]

Table S1: Food groups, number of food items per food group and flavonoids status

	N	N not containing flavonoids	N containing flavonoids
Tea	4	1	3
Fruits, nuts and seeds	163	70	93
Alcoholic beverages	138	65	73
Fruit and vegetable juices	11	7	4
Chocolate confectionery	64	52	12
Sugar, honey, jam, syrup	63	60	3
Dairy products and substitutes	367	356	11
Vegetables	159	83	76
Coffee and herbal teas	31	22	9
Legumes	18	12	6
Potatoes and other tubers	19	11	8
Condiments, spices and sauces	218	181	37
Meat, meat products and substitutes	298	292	6
Carbonated soft isotonic drinks	25	23	2
Cereals and cereal products	228	218	10
Fats and oils	50	49	1
Miscellaneous	19	18	1
Confectionery non chocolate	46	46	0
Mixed dishes based on cereals	137	137	0
Based on fish and crustaceans/mollusks	33	33	0
Based on meat and meat products	70	70	0
Based on vegetables (including potatoes)	59	59	0
Cakes and sweet biscuits	292	292	0
Eggs and egg products	16	16	0
Fish, shellfish	137	137	0
Salty snacks	105	105	0
Soups	23	23	0
Others non alcoholic beverages	10	10	0
Waters	32	32	0

Table S2: Distribution of tea flavonoid intake percentiles. Data for the total adult (N = 2121) and children (N = 1775) population.

		P5	P15	P25	P50	P75	P85	P95
Adults	All	0.0	0.0	0.0	0.0	74.2	210.8	473.3
Gender	Men	0.0	0.0	0.0	0.0	0.0	117.1	436.4
	Women	0.0	0.0	0.0	0.0	154.3	281.2	549.3
Age	18-44 yo	0.0	0.0	0.0	0.0	64.5	190.4	473.3
	45-64 yo	0.0	0.0	0.0	0.0	108.8	225.1	476.1
	65-79 yo	0.0	0.0	0.0	0.0	68.0	222.2	514.3
ICU	<900 €/m/CU	0.0	0.0	0.0	0.0	0.0	129.1	462.8
	[900-1 340[€/m/CU	0.0	0.0	0.0	0.0	54.4	190.4	436.4
	[1 340-1 850[€/m/CU	0.0	0.0	0.0	0.0	131.5	229.9	491.0
	>=1 850 €/m/CU	0.0	0.0	0.0	0.0	120.8	258.1	494.3
	Unknown	0.0	0.0	0.0	0.0	111.6	369.9	598.4
SPC	Low	0.0	0.0	0.0	0.0	37.6	138.8	367.0
	Medium	0.0	0.0	0.0	0.0	129.4	316.0	518.8
	High	0.0	0.0	0.0	0.0	168.9	360.8	504.4
	Not Working	0.0	0.0	0.0	0.0	42.5	180.7	473.2
	Doesn't know ; No answer	0.0	0.0	0.0	0.0	0.0	0.0	0.0
Education	Primary & middle school	0.0	0.0	0.0	0.0	0.0	114.1	393.4
	High school	0.0	0.0	0.0	0.0	102.0	229.7	480.0
	1 to 3 years of post-secondary education	0.0	0.0	0.0	0.0	168.1	316.0	515.9
	4 or more years of post-secondary education	0.0	0.0	0.0	0.0	172.8	364.9	562.8
	Unknown	0.0	0.0	0.0	0.0	0.0	0.0	0.0
BMI	Thinness	0.0	0.0	0.0	0.0	277.7	504.4	560.5
	Normal	0.0	0.0	0.0	0.0	118.9	263.5	508.2
	Overweight	0.0	0.0	0.0	0.0	54.4	167.8	462.8
	Obesity	0.0	0.0	0.0	0.0	0.0	170.6	363.4
	Morbid obesity	0.0	0.0	0.0	0.0	0.0	54.4	270.8
Children	All	0.0	0.0	0.0	0.0	0.0	0.0	0.0

Table S3: Distribution of tea flavonoid intake percentiles. Data for adult tea drinkers (N = 758) and children tea drinkers (N = 83) only.

		P5	P15	P25	P50	P75	P85	P95
Adults	All	30.9	63.5	84.9	199.4	393.4	477.2	598.4
Gender	Men	27.2	54.4	73.7	167.8	373.3	463.4	560.3
	Women	37.6	64.5	103.1	215.4	396.7	514.3	608.3
Age	18-44 yo	33.6	63.5	78.2	190.4	380.8	476.1	581.9
	45-64 yo	30.9	54.4	97.8	202.2	393.4	463.8	739.6
	65-79 yo	22.4	63.5	102.2	222.2	462.8	552.1	636.3
ICU	<900 €/m/CU	37.6	54.4	78.2	190.4	420.3	477.2	560.5
	[900-1 340[€/m/CU	21.5	54.4	79.3	202.6	433.1	439.2	562.8
	[1 340-1 850[€/m/CU	50.4	68.0	102.0	196.8	351.4	473.2	762.6
	>=1 850 €/m/CU	30.9	60.2	91.9	182.8	399.4	480.0	595.1
	Unknown	30.9	54.4	108.8	251.9	514.3	598.4	598.4
SPC	Low	21.5	63.5	79.3	190.4	269.2	458.6	560.5
	Medium	30.9	54.4	78.16	216.5	436.4	502.2	762.6
	High	51.6	74.2	114.6	205.1	420.0	504.4	598.4
	Not Working	22.4	59.2	76.6	190.4	376.5	492.1	636.2
Education	Primary & middle school	19.8	52.1	66.1	190.4	360.8	436.4	608.3
	High school	30.9	68.0	113.6	206.4	458.6	480.0	658.9
	1 to 3 years of post-secondary education	30.9	64.5	78.2	214.9	380.8	504.4	574.4
	4 or more years of post-secondary education	54.4	74.2	122.5	182.8	445.6	506.6	673.3
BMI	Thinness	53.5	224.4	371.6	560.5	566.1	622.0	651.3
	Normal	76.2	160.0	202.1	379.0	546.8	653.4	844.7
	Overweight	86.8	133.5	168.6	335.2	471.4	625.5	923.9
	Obesity	54.8	118.1	179.6	217.6	535.8	625.2	838.1
	Morbid obesity	91.2	133.4	145.1	250.7	324.8	383.3	551.7
Children	All	34.1	43.4	54.4	75.3	153.8	243.7	312.5
